# Calcific myonecrosis and the role of imaging in the diagnosis: A case report

**DOI:** 10.1080/03009730903016050

**Published:** 2009-09-07

**Authors:** Atsushi Okada, Masahito Hatori, Masami Hosaka, Munenori Watanuki, Eiji Itoi

**Affiliations:** ^1^Department of Orthopaedic Surgery, Tohoku University School of Medicine; ^2^Department of Orthopaedic Surgery, South Miyagi Medical Center

## Abstract

Calcific myonecrosis is a rare posttraumatic complication characterized by the replacement of muscles of one or more compartments with central liquefaction and peripheral calcification. We report magnetic resonance imaging (MRI) and CT imaging features of calcific myonecrosis arising 43 years after trauma. A 62-year-old man presented with a slowly enlarging mass in the left lower leg. Plain radiographs revealed a soft tissue mass with extensive amorphous calcification. The middle third of the tibia and fibula were eroded. MRI demonstrated peripheral ring enhancement on postcontrast fat-suppressed T1-weighted images. Volume-rendered images extracting only the images of bone and vessels also showed many branches from the tibialis posterior and peroneal arteries around the bone defect. During the operation, bleeding continued heavily from the vessels penetrating the cortical bone of the tibia, from the posterior compartment, and from the branches of tibialis posterior artery. The total blood loss was approximately 2,400 milliliters. There may be a causal relationship between massive bleeding and the hypervascularity of this tumor as evidenced by MRI and volume-rendering CT studies.

## Introduction

Calcific myonecrosis is a condition characterized by the latent formation of a dystrophic calcified mass occurring exclusively in the lower extremity (1). This rare condition has been reported to occur 10 to 64 years after the initial injury and typically presents as an enlarging mass in the anterior compartment of the leg. To date, 40 cases have been reported in the literature (1–23). Although the pathophysiological mechanisms associated with this rare disorder are not fully elucidated, several studies have suggested that this lesion is a late sequela of compartment syndrome (20,23), injury to the common peroneal nerve (18,19). Complete debridement is the most commonly recommended treatment for calcific myonecrosis (3). Although this treatment is often followed by severe bleeding, the cause of bleeding has not been made clear (10,16). There have been few reports concerning meticulous enhancement evaluation following contrast administration on either CT or magnetic resonance imaging (MRI) (9,10). To the literature on this rare condition is added a case report of a calcific myonecrosis arising in the lower leg with an emphasis placed on its radiological features of enhancement MRI and volume-rendering CT in order to observe the blood-stream to this rare tumor-like lesion.

## Case report

**Figure 1.**
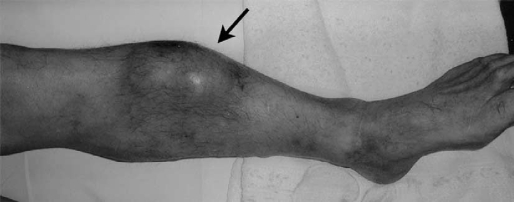
Mid-portion of the calf viewed from the anterior aspect. A bulging, non-tender and elastic hard mass was covered with the thin and atrophic skin.

**Figure 2.**
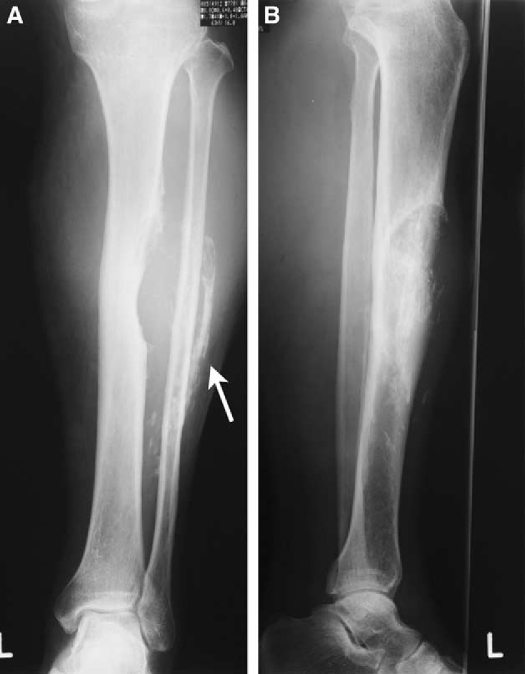
Plain radiographs of the left leg revealed a soft tissue mass in the anterolateral part of the leg with extensive amorphous calcifications (arrow).

**Figure 3.**
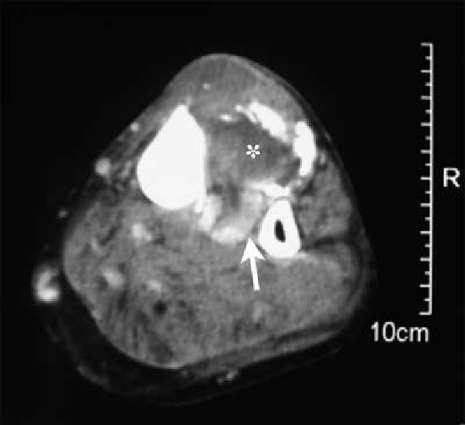
Postcontrast CT showed a 4×6 cm sized mass with central low signal density (asterisk) and peripheral high density, suggesting fluid and peripheral calcification. The space between the tibia and fibula was also enhanced (arrow).

**Figure 4.**
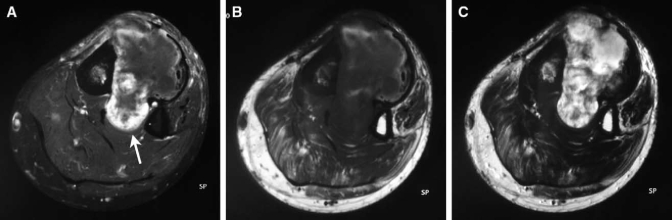
Magnetic resonance imaging demonstrated that the contents of the masses were isointense with muscles on T1-weighted images and hyperintense on T2-weighted images, with peripheral low signal intensity foci representing calcification. Postcontrast fat-suppressed T1-weighted image showed peripheral ring enhancement (arrow).

**Figure 5.**
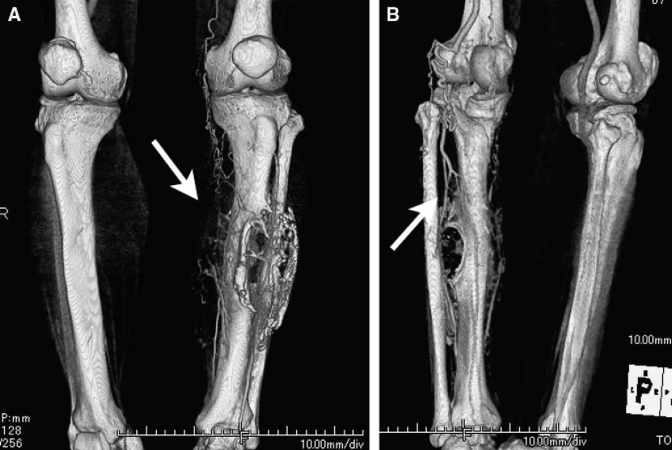
Volume-rendered image extracting only the images of bone and vessels showed many branches from the tibialis posterior artery and peroneal artery around the bone defect.

A 62-year-old man presented a 5-month slowly enlarging mass in the anterior part of the left lower leg. Forty-three years earlier, he had his left lower leg squeezed between two rollers during his work, and the popliteal vessels were injured. At the initial examination, there was no clinical evidence of a compartment syndrome. Since this accident, he noticed that the left foot showed some weakness at dorsiflexion. Physical examination showed a bulging, non-tender and elastic hard mass covered with thin and atrophic skin on the mid-portion of the anterior aspect of the left calf (Figure 1). The mass was sized approximately 13×9×5 cm. The circumference of the calf was 45 cm compared with 43 cm on the contralateral side. There was hypesthesia on the lateral side of the calf. Active dorsal flexion of the ankle and big toe was impossible. Plain radiographs of the left leg revealed a soft tissue mass in the anterolateral part of the leg with extensive amorphous calcifications (Figure 2). The middle third of the tibia and fibula was eroded. Postcontrast CT showed a 4×6 cm sized mass with central low signal density and peripheral high density, suggesting fluid and peripheral calcification. The space between the tibia and fibula was also enhanced (Figure 3). MRI demonstrated that the contents of the masses were isointense with muscles on T1-weighted images and hyperintense on T2-weighted image, with peripheral low signal intensity foci representing calcification. Postcontrast fat-suppressed T1-weighted imaging showed peripheral ring enhancement (Figure 4). We also applied volume-rendered imaging, extracting only the images of bone and vessels, which showed many branches from the tibialis posterior and peroneal arteries around the bone defect (Figure 5). Open biopsy revealed brown, semi-liquid tissues with interspersed calcification. Histological examination revealed necrotic tissues of skeletal muscles, dense fibroses with invasion of inflammatory cells. There was no evidence of malignancy. Bacterial culture was negative. After the biopsy, the mass enlarged gradually and discomfort appeared around the mass.

Six months after the biopsy, for the purpose of relieving pain, we performed marginal excision using a tourniquet. Surgical exploration through an anterolateral incision revealed a cystic cavity filled with brown-colored fluid with necrotic muscle debris. The cyst was surrounded partially by thin bone shells. The middle portion of the tibia was eroded, and there was a small area of cortical erosion involving the medial aspect of the fibula. Although the cystic cavity was attached to the bone erosion, it could easily be peeled from the bone. The cyst, the necrotic muscles, and calcification were removed. The surgical site of the anterior aspect of the left lower leg demonstrated a large deficit by resection of calcified tissue. Blood continued heavily from the vessels penetrating the cortical bone of the tibia, from the posterior compartment, and from the branches of the tibialis posterior artery. The total blood loss was approximately 2,400 milliliters. The wound was closed after placing a suction drain tube in the deep portion of the wound, and a bulky compressive dressing was applied to the lower leg. Postoperatively, the wound healed without infection. At 7-month follow-up, the patient was able to walk without pain. The muscle strength and sensory of his lower leg were normal.

## General concept of calcific myonecrosis

Calcific myonecrosis was first reported by Gallie and Thompson in 1960 (23). This entity is characterized by the replacement of muscle of one or more compartments with central liquefaction and peripheral calcification, resulting in a central cystic mass in the muscle. Of 40 cases reported in the English literature all but 1 case have a history of remote trauma, from 10 to 64 years after the initial injury (1). In the present case, the diagnosis was made based on the radiographic findings along with the remote history of trauma of 43 years ago. Calcific myonecrosis may be the continuum of pathologic processes including posttraumatic cyst of soft tissue, or chronic expanding hematoma (4). Mantzel et al. suggest that the similar clinicopathological entities of calcific myonecrosis, chronic expanding hematoma, posttraumatic cyst of soft tissue should all be considered as a single entity under the broad term ‘ancient hematoma’ (24).

## Differential diagnosis of calcific myonecrosis

The differential diagnosis of calcific necrosis includes malignant tumors with the propensity to mineralize, such as synovial sarcoma and soft tissue osteosarcoma (8,10,16,25), myositis ossificans, posttraumatic pseudoaneurysms, dermatomyositis, polymyositis, diabetic myonecrosis, and tumoral calcinosis (4,26,27). Soft tissue sarcomas can be distinguishable from calcific myonecrosis because the mineralization tends to be distributed throughout the tumor and not predominantly in the peripheral location. Myositis ossificans can be distinguished by the relatively short history after the injury in terms of weeks or months. In cases of dermatomyositis, polymyositis or diabetic myonecrosis, there is extensive calcification, but the remote history of trauma is lacking, and the systemic manifestation should be evident. Tumoral calcinosis is a rare clinical or histopathological syndrome that causes the formation of calcium deposits in the periarticular soft tissue, which is commonly associated with chronic renal failure. The history of renal disease and the fact that the location of calcifications is usually periarticular prevents it from being misdiagnosed as calcific myonecrosis (27). At first visit of the case, we suspected a malignant sarcoma such as osteosarcoma or synovial sarcoma because radiographs showed massive bone destruction with soft tissue mineralization. Histological examination of the open biopsy specimen demonstrated abundant acellular eosinophilic material mixed with necrotic muscles, granulofibromatic tissues. There was no evidence of malignancy. These findings were interpreted as consistent with myonecrosis. Taking into consideration the peripheral calcification and the past remote history of trauma, we diagnosed this condition as calcific myonecrosis.

## Radiological findings

Calcific myonecrosis has distinct radiographic features. The first one is the smooth pressure erosion affecting the outer cortex of the adjacent bone. It represents a chronic nature of this disease. The second is calcification of soft tissue, which is thin with a plaque-like or linear configuration organized around the margin of the lesion (3). Contrast-enhanced CT clearly shows the rim-like distribution of calcification with central homogeneous density, suggesting fluid retention. On MRI, a heterogeneous or homogeneous signal intensity mass has been reported on T1 and T2. The difference in signal intensity may depend on the degree of diversity of contents (2). There have been three reports concerning enhancement evaluation following contrast administration on MRI. O'Keefe et al. reported that the calcific myonecrosis mass was not enhanced with gadolinium on MRI study (16). Zohman et al. also reported that T1-weighed MRI made after the administration of gadolinium showed a lack of enhancement of the mass (10). Only Tuncay's group reported that peripheral rim enhancement was observed in a mass arising 42 years after a gun-shot (9). In our case, calcification was observed in the posterolateral portion of the mass on plain X-ray. The mass had a low-density area with peripheral calcification on CT scan with no cortical bone destruction. These findings are similar to the reported features of calcific myonecrosis (1,2,6,7,12). The mass showed heterogeneous enhancement on the fat-suppressed T1-weighted MRI, which may imply the hypervascularity of the tumor. Volume-rendered images can depict soft tissue and 3D relationships. It is useful in anatomic regions where vessels and bones are in close proximity (28). Also in our case, it clearly showed the relationship between vessels and bones. Small branches from the winding popliteal artery ran down and formed anastomosis around the tumor.

To the best of our knowledge, this report is the first to describe the anatomical relationship of the vessels with calcific myonecrosis.

## The cause of calcific myonecrosis

The exact pathophysiology of calcific myonecrosis has not been elucidated, but neurovascular compromise has been suggested to be the primary cause. It mostly occurs after tibial or fibular fracture resulting in a compartment syndrome. O'Keefe et al. hypothesized that an initial compartment syndrome decreases the circulation within a limited space resulting in necrosis and fibrosis (16). They speculated that the mass might enlarge because of repeated intralesional hemorrhage with time. Calcific myonecrosis can also occur after common peroneal nerve injury (15,19,22). The causal relationship between the common peroneal nerve injury and calcific myonecrosis, however, has not been elucidated. In our case, the patient did not have the history of compartment syndrome, but had a palsy of tibialis anterior and extensor hallucis longus which suggests the presence of common peroneal nerve palsy*.* In general, after contusion injury, skeletal muscle is remodeled often with fibrosis, not with necrosis (29–31). We hypothesize that blood-flow to the muscle was insulted because contusion injury to the muscle and injury to the popliteal artery took place at the same time, which led to myonecrosis. With time the mass expanded because of repeated intralesional hemorrhage supplied from the collateral arteries.

## Treatment for calcific myonecrosis

Treatment for calcific myonecrosis is controversial. With regard to the reported cases, excision has commonly been performed (2,13,16,17,23). However, several investigators recommend observation, because calcific myonecrosis is a benign entity and surgical intervention is often followed by secondary infection and chronic fistula formation (3,8,10,19,21). Blood loss is also a serious problem (10,16). In our case, we predicted preoperatively hypervascularity around the mass according to the enhanced MRI and volume-rendering CT. Our patient lost 2,400 mL blood. There may be a causal relationship between massive blood loss and hypervascularity of the mass as evidenced by the enhancement in the MRI study.

The diagnosis of calcific myonecrosis must be considered in cases with a history of remote trauma and the presence of calcific mass. Excision is thought to be the treatment of choice if the patient has pain and especially if imaging studies show no evidence of hypervascularity.
